# Role of Information Technology in COVID-19 Vaccination Drive: An Analysis of the COVID-19 Global Beliefs, Behaviors, and Norms Survey

**DOI:** 10.7759/cureus.15922

**Published:** 2021-06-25

**Authors:** Debjyoti Talukdar, Kire Stojkovski, Daniel B Suarez, Madan Mohan Gupta

**Affiliations:** 1 College of Pharmacy, Teerthanker Mahaveer College of Pharmacy, Moradabad, IND; 2 Sports Medicine, Faculty of Medical Science, Goce Delčev University of Štip, Shtip, MKD; 3 Integrative Medicine, Urbanización Trigal Centro, Valencia, VEN; 4 School of Pharmacy, Faculty of Medical Sciences, The University of the West Indies at St. Augustine, St. Augustine, TTO

**Keywords:** covid-19 vaccine survey, vaccine, sars ncov-2, covid-19, information technology, mit vaccine survey

## Abstract

With the onset of the COVID-19 pandemic, information technology has played a critical role in healthcare. A broad spectrum of information technology tools and applications played an essential role to create awareness of the COVID-19 vaccination drive and its health benefits. Research conducted by Massachusetts Institute of Technology (MIT) in collaboration with information technology platforms like Facebook with inputs from World Health Organization (WHO), John Hopkins University (JHU), and Global Outbreak Alert and Response Network (GOARN) shows that 65.06% of people all over the globe are willing to get vaccinated. Vaccine acceptance depends upon social norms and human behavior. These organizations conducted the global survey in over 60 countries with a sample size of 437,236 responses. The international survey was organized using a pre-registered randomized experiment demonstrating the role of technology in reaching out to people based in diverse communities and evaluating their beliefs, behavior, and social norms. The study shows that vaccine acceptance can vary due to descriptive norms. All the respondents in the study were adults with access to the internet. Moreover, a large proportion of the population thinks that the COVID-19 pandemic is a viable threat to the community and preventive measures need to be taken including vaccination drives to eradicate the menace. The survey consisted of five blocks involving questions related to healthcare, demographics, vaccines, knowledge, and information exposure. Sampling and weighting were done using a pool of 3,000 respondents over two weeks, and weights were provided per respondent to represent the target population as a whole. It reduces the representation error and minimizes non-response biases.

## Introduction

A global survey was conducted using information tools to study the norms, beliefs, and human behavior toward COVID-19 and vaccination drive. It was conducted by Massachusetts Institute of Technology (MIT), Facebook, and input from various institutions like World Health Organization (WHO), John Hopkins University (JHU), and Global Outbreak Alert and Response Network (GOARN). Information technology allows us to monitor the vaccination drive, create awareness, and develop a technical framework to better respond to challenges arising to the global health system [1-5].

The study conducted by these organizations allows measuring the behavioral response of the participants toward the vaccination drive. It involves critical information related to the availability of multiple vaccines, hesitancy toward vaccines, and behavioral norms toward vaccine acceptance [6-9]. Country-wise statistical information shows the effectiveness of information technology toward improving the behavioral response of the public to accept vaccination and achieving herd immunity. Though there are lots of barriers toward imposing the vaccination drive such as false beliefs and misconceptions, technological tools like online surveys conducted by Facebook have the potential to reach over two billion people around the world. It has partnerships with numerous academic and nonprofit institutions to offer humanitarian efforts and tackle challenges about the COVID-19 vaccination drive [10-14].

The COVID-19 survey has the potential to reach millions of internet users worldwide. It includes demographic information regarding country of origin, gender, age group, and education. It can reveal critical information regarding early warning, epidemic detection, public awareness, vaccination acceptance, beliefs, and control mechanisms. The framework of the survey offers technical insights about managing the outbreak; reviewing health-specific information; highlighting the importance of vaccination and relevant challenges related to technology design, implementation, and use [1-2,15-18].

The study comprises 437,236 participants from 60 countries, conducted by MIT, JHU, and WHO. It involves risk perception and preventive behavior among adults aged 20-80-years old. The study involves significant predictors like knowledge about vaccination drive, local insights, public health decisions, and communication. The study conducted by MIT and Facebook is fully secure as it offers complete privacy to the respondent and valuable information regarding understanding the importance of vaccination drive and sharing knowledge and practices.

There are numerous benefits regarding the survey conducted by MIT and Facebook as it will provide policymakers with critical insights to understand the effectiveness of the vaccination drive, preventive behavior, and norms of the public globally [1-2,19-22]. It includes awareness of the healthcare policies about wearing masks and social distancing along with parameters to measure the effectiveness of communication between public health officials and the global community. Moreover, researchers can benefit from this survey as well as can request access to the data and study the behavioral perception of the community toward vaccination and understand areas that need improvement. It also involves population characteristics like level of education, age, gender, geographical location, etc., which will benefit researchers to understand the risk of an outbreak [23-25].

The aim of this study is to use the primary data collected by MIT, Facebook, JHU, and WHO to research and interpret through statistical tools and provide information on how technology is useful in vaccination drives.

## Materials and methods

**Figure 1:**
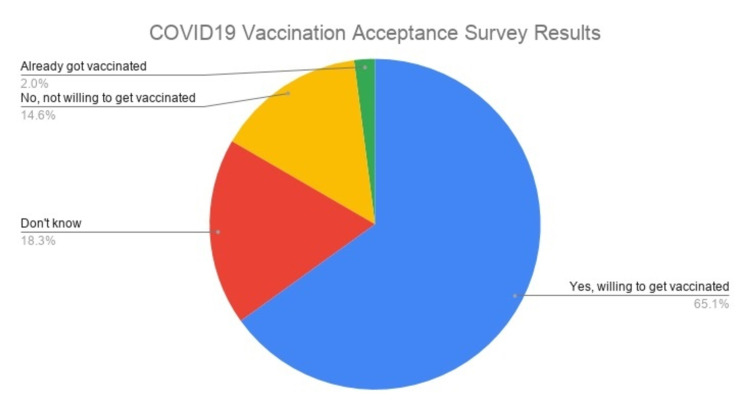
COVID-19 Vaccination Acceptance Survey Results of 437,236 Participants From 60 Countries

The study was done based on the primary data (aggregated data Application Programming Interface [API]) freely available for researchers worldwide through the Creative Commons Attribution International License. The global survey on coronavirus belief, behavior, and norms was conducted by MIT in collaboration with Facebook and researchers from JHU, WHO, and the GOARN that involved a sample size of 437,236 participants from 60 countries worldwide (Figure 1) [1,2].

Study design

The survey data was analyzed using descriptive statistics that involve one or more variables and how they are related to each other. Frequencies for categorical variables were calculated such as those who are willing to get vaccinated, not willing to get vaccinated, already got vaccinated, and don’t know. The frequencies offer an overall view of several observations in each category. The study also involves summary statistics as percentile offers us an overall view of how the data is distributed. The distribution of the data across various countries shows that it is not symmetric as the percentile of the population who are willing to get vaccinated, already got vaccinated, don’t know, and not willing to get a vaccination in each respective country. Also, the sample size for each country is asymmetrical as well. Summary statistics were conducted with one quantitative variable over three categorical variables. The observations for each categorical variable show how each variable is related to the other.

The survey was analyzed using STATA (statistical software with graphical visualization of data, StataCorp, Texas, USA). STATA tab and sum commands were involved in the analysis of the data. Percentages were added using the tab output command. Other STATA commands like row and column were also used to compute percentages for each variable. The summarize command creates a statistical summary by creating a table for the single quantitative variable. The distribution of the data for various countries was compiled and analyzed through STATA commands incorporated through the.dta dataset. The histogram tool used in the analysis of the data helps us to understand its distribution. The respondents in the survey come from diverse backgrounds, education levels, ethnicity, gender, and age. The data was organized in the form of rows and columns in order to understand the overall behavioral views of the group of population toward the vaccination drives. The row represents the first variable, while the column represents the second variable. The row (various countries) represents the dependent variable that was analyzed in relation to the independent variables (willing to get vaccinated, not willing to get vaccinated, already vaccinated, and don’t know). STATA command sum shows the summary analysis for both dependent and independent variables represented in each cell of the table.

Explanatory variables

**Figure 2:**
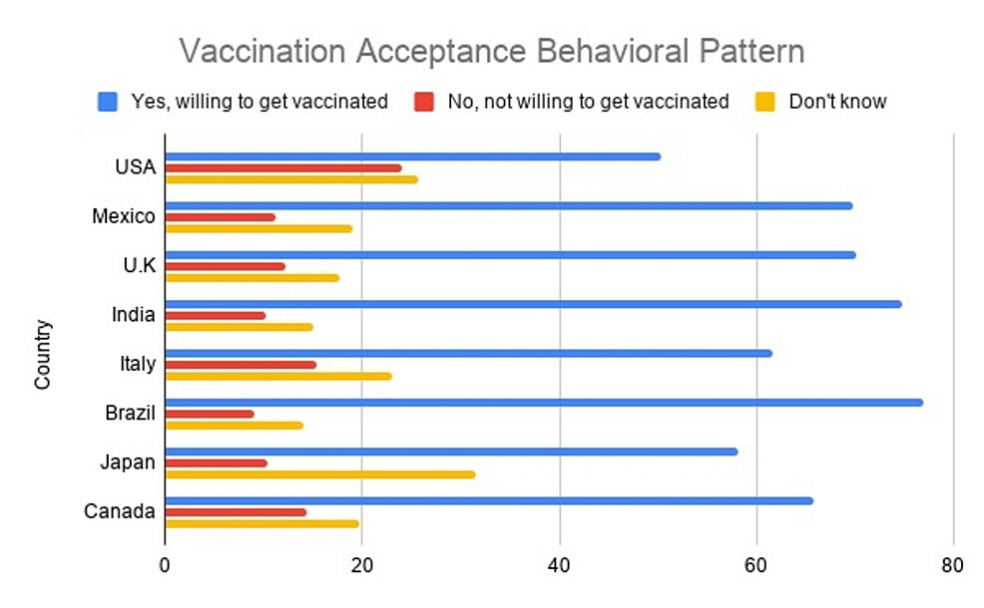
Country-wise Vaccination Acceptance Behavioral Pattern

The parameters used in the study were age, gender, education, race, and country. The variable age was divided into six categories 20-30, 31-40, 41-50, 51-60, 61-70, and 71-80, respectively. The gender category was subdivided into male, female, and other/unknown. The category education was subdivided into less than primary school education, primary school, secondary school, college-level education, and graduate education. The race category is subdivided into White, Hispanic, Asian, American Indian, Alaska Native, Black, African American, Hawaiian Native, Pacific Islander, and others. A two-letter ISO (International Organization for Standardization) code was assigned to 60 countries all over the globe. For example, US - United States of America, MX - Mexico, CA - Canada, BR - Brazil, and IN - India. Similarly, a two-letter code was used for the United States and Indian states, respectively (Figure 2).

The survey consisted of specific signals indicating various aspects of vaccination and preventive measures associated with the COVID-19 pandemic. Each signal name was assigned a unique variable categorized with a set of options. For example, the signal name "vaccine accept" indicates whether the respondents are willing to get vaccinated if a COVID-19 vaccine becomes available. The output values consisted of options like Yes, Don’t Know, or No. Another signal name "norms_vaccine" asked the respondents to approximate the number of people who will take the vaccine out of 100 if it was made available in a community. The signal name "density" describes the area where they are currently based. Another signal name "flu_vaccine" indicated whether the participants received their flu vaccine or they plan to take one in the upcoming weeks. The answer choices were subdivided into “Don’t know,” “No,” and “Yes.” Signal name "future_vaccine" showed that if a certain vaccine was available in the market, are they willing to take it? The option choices were “No, definitely not,” “Probably,” “Probably, not,” “Unsure,” and “Yes, definitely.” Another signal "future_vaccine_recommended" asked the respondents whether they are willing to take a vaccine against COVID-19 infection if it was made available and recommended to them by the following: (1) “family and friends” and (2) “government health officials” with answer choices “Less Likely,” “More likely,” or “No impact.”

The survey was analyzed using regular regression and post-stratification methods. It involved predictive analysis and health informatics with the help of Farr Institute (https://www.farrinstitute.org/healthcare-informatics) wherein complex and adaptive modeling techniques were used for large-scale data mining, statistics, and analysis. The study also involved a set of weights for the survey participants, modeling, assessing the pattern of engagement for each respondent including response and non-response behavior along with attributes for each participant. The study also involved randomized checks to build a robust dataset.

## Results

The survey conducted across 60 countries with 437,236 participants shows that 65.06% of the respondents are willing to get vaccinated with 2.03% of the total respondents already vaccinated; 18.03% of the respondents stated that they were not sure about vaccination, while 14.60% of the respondents stated that they don’t want to get vaccinated. The results were computed in waves with a two-week duration period for each wave. The time frame for the first wave started from July 06, 2020, to July 19, 2020; subsequent waves continued till March 28, 2021.

In terms of behavioral belief and norms toward the COVID-19 pandemic, 21.28% of all the respondents consider it extremely dangerous to the community, while 6.37% of the respondents consider it as not dangerous at all; 23.67% of the respondents consider the pandemic as moderately dangerous, and 14.09% of the respondents consider it as slightly dangerous. Given the community outbreak and risk of contracting the virus at public places of engagement, 45.97% of the respondents indicated that they would prefer to visit retail shops amid the COVID-19 pandemic, 42.40% would prefer to visit healthcare facilities, and 40.44% of the respondents would visit places of employment.

In the United States, 50.33% of the respondents are willing to accept vaccination, 25.70% of the respondents were not sure about the vaccination, and 23.96% don’t want to get the vaccination. In Mexico, the vaccine acceptance rate is higher than that in the United States as 69.71% of the respondents are willing to take the vaccination, 19.09% of the respondents are not sure, and 11.18% of the respondents are ready to accept the vaccination. In India, 74.77% of the respondents are willing to accept vaccination, 14.99% of the respondents are not sure, and 10.23% of the respondents are not willing to accept vaccination (Table 1).

**Table 1:** Country-Specific Vaccination Acceptance Behavioral Pattern and Sample Size

Country	Yes, willing to get vaccinated	No, not willing to get vaccinated	Don't know	Sample size
USA	50.33	23.96	25.7	15,675
Mexico	69.71	11.18	19.09	15,593
U.K	70.08	12.27	17.63	15,391
India	74.77	10.23	14.99	10,114
Italy	61.63	15.3	23.06	19,742
Brazil	76.96	8.99	14.03	20,516
Japan	58.2	10.33	31.46	10,942
Canada	65.83	14.45	19.71	4,119
Australia	73.42	10.75	15.81	4,334
Nepal	76.23	9.23	14.53	1,530
Algeria	42.08	29.68	28.23	2,545
Afghanistan	77.72	9.61	12.66	1,145
Mozambique	61.43	11.3	27.25	3,107
Cameroon	35.05	42.18	22.76	3,659
Morocco	44.32	25.4	30.27	2,211
Sri Lanka	65.64	15.26	19.09	1,486

## Discussion

Information technology plays a critical role in raising awareness among the general public about the importance of vaccination against COVID-19 infection. The severe acute respiratory syndrome novel coronavirus-2 (SARS-nCoV-2) is an infectious disease, and it can make us sick once we are exposed to the virus. A vaccine combats the spread of the infection in our body by activating the immune system and preventing further harm efficiently. COVID-19 virus has the potential to harm us as we are constantly exposed to the virus while visiting public places like restaurants, retail stores, businesses, places of worship, public parks and gardens, and places of work. Exposure to the virus can trigger our immune system to react to the virus as it has high negative outcomes toward our body, which has led to intense research and human clinical studies about effective COVID-19 vaccination [20-29].

Mass production and distribution of reliable SARS-nCoV-2 virus require the use of information technology. Study shows that vaccination offers optimal protective immune response against the virus. It is seen that COVID-19 patients suffered disease severity to a greater extent with hospitalization and high-intensity medical care. The beneficial effects of the COVID-19 vaccine can be evaluated through seroconversion as multiple trial strategies encompassing individuals from diverse backgrounds were recruited to compare and validate serological assays. Presently, we have various vaccine products from different clinical trials. Information technology played a critical role in changing behavioral patterns and increasing the number of enrollees for vaccination. It is important to harmonize the various results of COVID-19 vaccination trials. Vaccination acts as a preventive measure against the spread of the COVID-19 infection. It reduces the risk of contracting the SARS-nCoV-2 virus. The primary goal of vaccination trials is to make sure that the subsequent SARS-nCoV-2 infection risk remains low and the vaccine is safe to administer. The vaccine trial shows that it is efficient in neutralizing the virus with the enhancement of the antibodies [25-29].

Information technology has helped in formulating coordinated efforts in accelerating the development of the vaccination program with institutions like the WHO, US Food and Drug Administration (FDA), Centers for Disease Control and Prevention (CDC), European Medicines Agency (EMA), and US National Institute of Health (NIH) along with several other organizations from across the globe. It has played a crucial role in coordinating the efforts between several research groups and biopharmaceutical companies. Most importantly, it has built a platform using various tools toward collaborative efforts between various organizations like Accelerating COVID-19 Therapeutic Interventions and Vaccines (ACTIV) in prioritizing various drug and vaccination clinical trials, streamlining candidates for vaccination, building assets, and a consortium of healthcare professionals to respond against the COVID-19 pandemic [1-2,14,24,28].

In the survey study, the respondents were shown how previous participants have answered survey questions in their country regarding behavioral perception about COVID-19 pandemic; vaccination drive; social distancing; preventive measures like wearing masks, hand washing, using sanitizers, etc. [15,22-28]. The information to the respondents was provided in a randomized way during the survey which showed the descriptive norms of the respondents about acceptance of the vaccination. The responses entered by the previous participants were summarized in a result format. Moreover, a message was shown at the beginning of the survey stating their responses are helping researchers across the world about the current COVID-19 pandemic and vaccination program. The weighted percentage was calculated as per responses received about measured outcomes of accepting the vaccination if it is made available to the respondents [16,23-25].

Limitations

The survey study has limitations in terms of wave time series as certain questions were asked to respondents from wave 9 (October 26, 2020, to March 28, 2021 instead of July 06, 2021, to March 28, 2021). Also, out of all 60 countries, the conditions of the United States and India were only included in the survey. The survey lacked vaccine efficacy studies for individuals who are already vaccinated. Extrapolation of vaccine data needs to be expanded beyond the geographically diverse population of more than 60 countries. The potential human challenges need to be highlighted and explored further. The role of public health authorities toward raising vaccine awareness and how it varies among various countries needs to be evaluated as well. Also, the population subgroup needs to be divided based upon the type of vaccine available in their respective country and its novelty. Overall, the survey was limited to global behavior, norms, and beliefs in relation to the current vaccination drive.

Ethical approval

We obtained the publicly available aggregated data from the MIT website (https://covidsurvey.mit.edu/api.html). The aggregated data API is freely available through the Creative Commons Attribution International License for various research groups. We have cited a global survey on coronavirus beliefs, behaviors, and norms in our article as required for data usage [1]. Our study is exempted from obtaining ethical approval as it consists of secondary data for research purposes.

Data availability

The data is freely made available by the MIT in collaboration with Facebook through aggregated data API from the COVID-19 Beliefs, Behaviors, and Norms Survey (https://covidsurvey.mit.edu/api.html).

## Conclusions

Information technology has played a critical role to control the outbreak of the COVID-19 pandemic and create awareness about vaccination drives. It has helped policymakers and researchers around the world to understand people’s behavioral patterns, norms, and beliefs about vaccination and combating the spread of the infection. The global survey conducted by MIT and Facebook in collaboration with JHO, WHO, GOARN, and other institutions offered a lot of insights at the respondent level of data regarding vaccination. The results show the importance of health information technology as a pivotal role to prevent the spread of the pandemic and increase development efforts concerning vaccination.
